# Global assessment of genomic variation in cattle by genome resequencing and high-throughput genotyping

**DOI:** 10.1186/1471-2164-12-557

**Published:** 2011-11-14

**Authors:** Bujie Zhan, João Fadista, Bo Thomsen, Jakob Hedegaard, Frank Panitz, Christian Bendixen

**Affiliations:** 1 Group of Molecular Genetics and Systems Biology, Department of Molecular Biology and Genetics, Faculty of Science and Technology, Aarhus University, Blichers Allé 20, DK-8830 Tjele, Denmark; 2 Department of Molecular Medicine (MOMA), Aarhus University Hospital, Skejby, Brendstrupgaardsvej 100, DK-8200 Aarhus N, Denmark

## Abstract

**Background:**

Integration of genomic variation with phenotypic information is an effective approach for uncovering genotype-phenotype associations. This requires an accurate identification of the different types of variation in individual genomes.

**Results:**

We report the integration of the whole genome sequence of a single Holstein Friesian bull with data from single nucleotide polymorphism (SNP) and comparative genomic hybridization (CGH) array technologies to determine a comprehensive spectrum of genomic variation. The performance of resequencing SNP detection was assessed by combining SNPs that were identified to be either in identity by descent (IBD) or in copy number variation (CNV) with results from SNP array genotyping. Coding insertions and deletions (indels) were found to be enriched for size in multiples of 3 and were located near the N- and C-termini of proteins. For larger indels, a combination of split-read and read-pair approaches proved to be complementary in finding different signatures. CNVs were identified on the basis of the depth of sequenced reads, and by using SNP and CGH arrays.

**Conclusions:**

Our results provide high resolution mapping of diverse classes of genomic variation in an individual bovine genome and demonstrate that structural variation surpasses sequence variation as the main component of genomic variability. Better accuracy of SNP detection was achieved with little loss of sensitivity when algorithms that implemented mapping quality were used. IBD regions were found to be instrumental for calculating resequencing SNP accuracy, while SNP detection within CNVs tended to be less reliable. CNV discovery was affected dramatically by platform resolution and coverage biases. The combined data for this study showed that at a moderate level of sequencing coverage, an ensemble of platforms and tools can be applied together to maximize the accurate detection of sequence and structural variants.

## Background

The domestic cow (*Bos taurus*) is a ruminant that belongs to the Cetartiodactyl order of eutherian mammals. Being phylogenetically distant from primates and rodents and with a drastically different biology, cattle serve a significant role as animal model for studies of evolution, metabolism, reproduction, and disease [1]. The recent publication of the cattle genome assembly and the insights into sequence and structural variations identified in the bovine HapMap project has sparked the full potential of cattle genomic research, expanding our knowledge of mammalian evolution and biology [2-5]. Furthermore, the integration of population-wide genotype information with phenotypic registrations generated in the dairy and beef industry provides an important resource for uncovering genes associated with complex production traits [6-10].

Despite the fact that SNP genotyping technology has enabled successful genome-wide association studies (GWAS) in humans and in livestock species [11-13], it has known disadvantages. For example, an ascertainment bias derived from the fact that the SNPs used are chosen to have a minimum "rare" allele frequency as well as to segregate in multiple breeds is sometimes introduced. The identification of rare causal mutations might be complicated due to failure to detect the disequilibrium between causal mutations and genotyped SNPs [14,15]. In contrast, whole genome sequencing of single individuals removes the polymorphism ascertainment bias, detects rare putative functional variants and also retrieves structural variants [16], an important category of genomic variation that only recently has become fully appreciated [17-24]. Despite their known functional importance in humans [21,23], so far only a few small-scale studies have probed the extent of structural variants, mainly copy number variation (CNV), in cattle [25-30]. The increasing cost efficiency of sequencing technologies has enabled large scale sequencing of individual genomes, which has dramatically increased of the catalogue of genomic sequences and structural variants detected and filled some of the earlier gaps in resources that were biased towards common sequence variants [24,31-43]. Therefore, cattle research initiatives similar to the human 1000 Genomes and Personal Genome projects [24,44] are of paramount importance in order to obtain a complete catalogue of genomic variation in this species. This catalogue will help researchers to efficiently associate genomic information with productivity traits and improve disease resistance to achieve breeding goals [45]. Two recent studies have reported the analysis of whole genome sequencing of cattle (Fleckevieh and Kuchinoshima-Ushi bulls) focussing on SNP discovery [46,47]. Nonetheless, to work out the links between DNA sequence and phenotype, efforts to sequence the genomes of more individuals are intensifying [48,49].

In this study, we have sequenced the genome of a Holstein-Friesian bull using massive parallel sequencing obtaining about 15 fold sequencing depth. Significant sequence and structural variations were found using an ensemble of variant callers: SNPs were identified using Mosaik+GigaBayes [50,51] (hereafter giga), CLC Genomics Workbench [52] (hereafter clc), BWA+SAMtools [53,54] pileup (hereafter bwa) and SMALT+SAMtools [54,55] pileup (hereafter smalt); intra read indels were detected with BWA+mpileup and BWA+Dindel; and inter read indels, inversions, and translocations were found using Pindel and BreakDancer [53,54,56-59]. Copy number variants were detected using three different platforms: sequence read-depth analysis; CGH arrays by signal intensity variation by comparing two samples; and SNP arrays by clustered pool references, signal intensity, and allelic intensity [29,60,61]. Sequence and structural variants were validated with the BovineHD and BovineSNP50 BeadChips (Illumina, San Diego, California, USA), and RT-PCR respectively [62]. The tools and pipelines applied here demonstrate that whole genome sequencing at relatively modest coverage levels is sufficient firstly, to survey sequence and structural variations by integrating different variation detection methods which minimized the false positive rate of polymorphism detection, a known problem of deep sequencing [63-66] and secondly, to provide accurate information across different classes of structural genetic variants.

## Results and Discussion

### Data production

**Figure 1:**
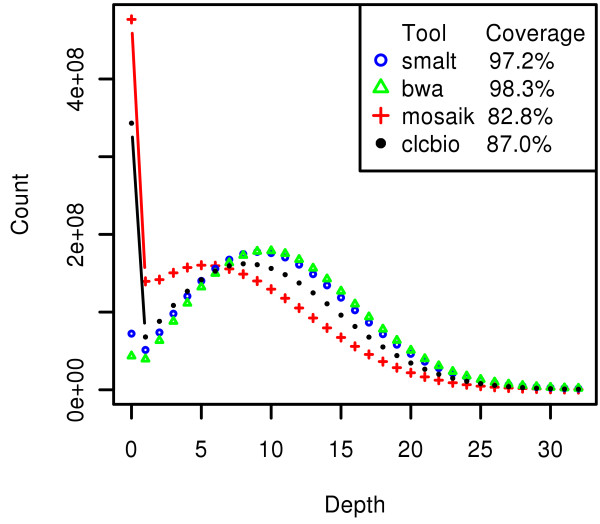
**Genomic coverage and read depth obtained after mapping**. Reads were mapped to the bovine genome assembly UMD3.1 using four different mapping tools independently. smalt, SMALT alignment tool; bwa, Burrows-Wheeler Alignment Tool; mosaik, Mosaik assembler; clcbio, CLC Genomics Workbench.

Massively parallel DNA sequencing using Illumina paired-end read chemistry [34] was performed to analyze sequence and structural genomic variation in a Holstein-Friesian bull. Two paired-end libraries with a span size of 300 and 500 bases were constructed, and 41 gigabases of sequence were generated (about 1.2 billion 36 base pair reads) resulting in a sequencing depth of about 15 fold. The sequences were aligned to the *Bos taurus *reference genome assembly UMD3.1 [67] with BWA [53]. After mapping on UMD3.1 and removing possible PCR duplicates, an average depth of 14.8 fold coverage was achieved; 98.3% of the reference genome (including chromosome unknown) was covered and 94.3% of the aligned bases had a phred-like quality score ≥20. Over 93.8% and 89.9% of the genome was covered by at least 3 and 4 reads, respectively (Figure 1). Approximately 3% of the reads were not mapped to the reference assembly, possibly because of a combination of breed or individual uniqueness, sequencing errors and contamination. All the sequences were also mapped to the UMD3.1 assembly using the CLC Genomics Workbench [52], Mosaik [50] and SMALT [55], and used mainly for SNP analysis comparisons. Figure 1 shows the mapped sequence depth variation for the different aligners (See also additional file 1). Uncovered bases were included when calculating depth. There were more uncovered bases (depth 0) with CLC and Mosaik because of the unique mapping strategy applied. Mapping onto the assembly Btau4.0 [2] using Mosaik produced similar results to those for the UMD3.1 assembly (Additional file 2).

### SNP detection

First, we identified SNPs and evaluated the performance of different SNP detection methods.

#### SNP detection based on resequencing

**Figure 2:**
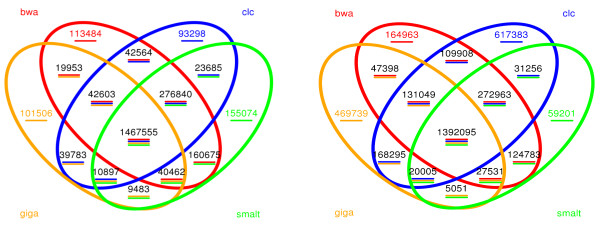
**The overlapping of SNPs called by four pipelines and based on the UMD3.1 reference assembly**. Left, homozygous; right, heterozygous. More than six millions unique SNP sites were identified by at least one of the aligners; 45.8% of them are consistent among all aligners. smalt, SMALT+SAMtools pipeline; bwa, BWA+SAMtools pipeline; clc, CLC Genomics Workbench; giga, Mosaik+GigaBayes pipeline.

The detection of small genetic variations was performed after mapping using the four aligning tools mentioned above (Table 1). For the UMD3.1 reference assembly, a total of 6,239,482 SNPs were called by the four pipelines combined, 1,774,648 SNPs were called by only one of the four pipelines, and 2,859,650 of the called SNPs were at the intersection of all four pipelines (Figure 2).

**Table 1:** SNPs called on the UMD3.1 assembly using different pipelines.

Tools	Algorithm	SNPs	Homozygous	Heterozygous
BWA + SAMtools	Bayesian *	4,434,826	2,164,136	2,270,690
CLC Genomics Workbench	NQS	4,740,179	1,997,225	2,742,954
SMALT + SAMtools	Bayesian *	4,077,556	2,144,671	1,932,885
Mosaik + GigaBayes	Bayesian	3,993,407	1,732,242	2,261,165

NQS, Neighbourhood Quality Standard; * pipelines that were implemented with mapping quality.

#### Functional analysis of SNPs

To exclude most of the false positive predicted SNPs from the functional analysis, we assessed possible functional effects only for the SNPs that were called by all four pipelines. Of these SNPs, 1,051,772 SNPs were located inside genic regions, including 27,722 coding SNPs of which 11,545 are predicted to cause either non-synonymous amino acid substitutions in proteins or to generate gain or loss of 182 stop codons. Eighty-one SNPs were located inside transcriptional splice sites, including 46 acceptor sites and 35 donor sites, and they have the potential to cause alternative splicing transcripts [68-70]. Using SIFT [71,72] and PolyPhen [72] to predict the effect of missense mutations, we found 286 homozygous and 530 heterozygous SNPs that were predicted to be deleterious to protein function. These missense SNPs correspond to 5% and 9.1% of the homozygous and heterozygous non-synonymous SNPs (nsSNPs), respectively; the difference reflecting the fact that deleterious alleles are less likely to be homozygous SNPs. However, we observed that the frequency of deleterious SNPs within CNV regions was 3.3-fold higher than the average across the whole genome (Chi-square test, p = 0.0001). The bias of deleterious SNPs in CNV regions may be explained either by a sequence duplication resulting in pseudogenes or by gene duplication where one copy harbours a deleterious mutation while the other retains the gene function. A gene set enrichment analysis for genes with nsSNPs using all bovine genes as background showed an enrichment bias for olfactory transduction and immune related pathways (p < 0.05). This result is similar to the result of the enrichment analysis of genes located inside CNV regions (see the discussion of the CNV regions below).

We found that the SNP frequency in the X chromosome is about 4.9 times lower than in the autosomes. Because, while autosomes are diploid, in the sequenced bull, the X chromosome is haploid; therefore, the sequencing depth of the X chromosome will be about half that of the autosomes resulting in the lower SNP detection rate. However, data in this study and in previous studies [35] have indicated an almost linear correlation between the SNP detection rate and sequencing depth in this sequence depth range, suggesting that low sequencing depth only partially explains the low SNP frequency found on the X chromosome. The low variant frequency observed on the X chromosome of the sequenced animal also suggests that cattle selection often favours a lower mutation rate on the X chromosome compared with on the autosomes because of the exposure of deleterious recessive mutations on hemizygous chromosomes [73,74].

We also found that the SNP frequency in CpG islands is approximately half that of the SNP frequency in the rest of the genome (Chi-square test, p < 0.0001) indicating that CpG islands are under more stringent selective pressure. Variants in CpG islands can potentially break the structure of the CpG dinucleotides thereby affecting the methylation status of the cytosine residues, possibly resulting in abnormal epigenetic regulation of gene expression.

Because of the limited number of imprinted genes that have been experimentally discovered in the cattle genome, we applied ortholog mapping of murine and human imprinted genes to the cattle genome based on Imprinted Gene Databases [75]. We found 47 cattle genes that were potentially imprinted and that the frequency of the heterozygous nsSNPs was 3.7 fold lower in the imprinted genes than in all the other genes in the genome (Chi-square test, p = 0.0048). This result suggested that the potentially imprinted genes underwent positive selection and is consistent with the parental conflict theory [76,77]. However, because the imprint status of these genes is yet to be determined experimentally in cattle, these results need to be verified by future studies.

Based on the pedigree information for the sequenced animal (Additional file 3), the inbreeding coefficient was calculated by Pedigraph v2.4 to be 0.046875 [78], suggesting that some identical by descent (IBD) regions exist in the genome. The SNPs detected in this study also provided a means to survey large IBD regions that were revealed as runs of homozygosity (ROH) in the sequenced animal. Here, we defined the ROH regions by applying a sliding window with size 1 Mb and a step size 200 Kb, with the ratio of heterozygous and homozygous SNPs set to less than 0.1. Using these criteria, 71 ROH ranging in size from 1 Mb to 92.4 Mb and corresponding to 13.5% of the whole genome in the sequenced individual, were identified.

Because the length of each IBD run is determined partly by the number of generations since the common ancestor, there is a continuum in the length of homozygous segments, depending on the degree of shared parental ancestry and its age. ROH due to recent inbreeding tend to be longer because there has been little opportunity for recombination to break up the IBD segments. On the other hand, ROH of much older origin are generally shorter because the chromosomal segments have been broken down by repeated meioses [79]. According to pedigree data from this study, the two largest IBD regions, with sizes of more than 50 Mb, were most likely to have been derived from a shared ancestor three generations ago.

### Genotyping with the SNP chips

In addition to whole genome sequencing, the Holstein-Friesian bull was genotyped using both the Illumina BovineHD and BovineSNP50 BeadChip arrays. After quality filtration, the arrays revealed 770,343 and 52,345 effective SNP genotypes, respectively. Of the 47,093 SNPs that were shared on the two SNP arrays, only 26 had different allele calls and they were mostly heterozygous/homozygous disagreement calls. If we assume that these 26 inconsistent alleles were incorrect genotypes, a SNP chip error rate of 26/47093 = 0.0552% can be deduced. Further filtering with unambiguous chromosome positions on the UMD3.1 assembly retained 756,243 and 42,603 effective genotypes for the BovineHD and BovineSNP50 arrays respectively. Excluding the heterozygous SNPs on the sex chromosomes, which, in a male genome, can arise either by error or within the pseudoautosomal region, left 755,397 polymorphic sites consisting of 207,670 heterozygous and 547,727 homozygous SNPs based on the BovineHD BeadChip results. After similar filtering, 42,587 SNPs consisting of 11,569 heterozygous and 31,018 homozygous SNPs remained based on the BovineSNP50 BeadChip results.

### Comparison of the SNP chip and resequencing results

Detection rate, accuracy and false positive rate (FPR) are crucial quality indicators that are affected mainly by the algorithm applied in the analyses pipeline and the depth of genome sequencing. The SNP detection rate in the whole genome resequencing data was calculated as the percentage of SNPs on the BovineHD BeadChip array that was also discovered in the resequencing data. The SNP detection accuracy was reported based on genotype consistency between the resequencing and BovineHD BeadChip data. A comparison of the results obtained in this study with the results published by Eck et al [46], considering only the sequence depth and disregarding the different pipelines that have been applied, revealed that an increase in sequence depth from 7.4 to 15.5 fold significantly improved both the accuracy and sensitivity of SNP calling. For example, the detection rate of SNP sites almost doubled from 42.98% to 80.0% and the accuracy increased from 70.67% to more than 93% (with slight variations for the different pipelines applied in this study).

**Figure 3:**
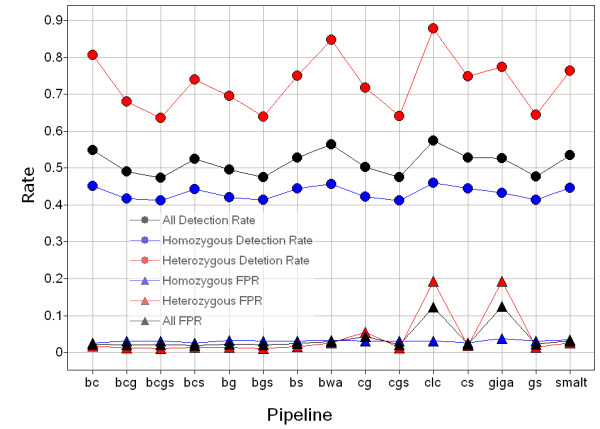
**Single and combined pipeline performances for SNP detection**. FPR denotes false positive rate, bwa denotes BWA+SAMtools, clc denotes CLC Genomics Workbench, giga denotes Mosaik+GigaBayes, smalt denotes SMALT+SAMtools, bc denotes the combination of bwa and clc, bcs denotes the combination of bwa, clc and smalt, bcgs denotes the combination of bwa, clc, giga and smalt. False positive rate for the heterozygous SNPs was calculated based on the heterozygous SNPs observed in an identical by descent region (IBD); the false positive rate for homozygous SNPs was calculated based on the homozygous SNPs observed in an IBD region in which the sequences are identical to the reference sequences. Pipelines without the implementation of alignment mapping quality parameter resulted in a higher FPR for heterozygous SNP detection.

The majority of SNPs were consistently called by both the BovineHD BeadChip and the resequencing pipelines; the inconsistent calls were separated into different categories: 1) heterozygous SNPs under-called as homozygous because of inadequate sequencing depth, or homozygous SNPs over-called as heterozygous caused by incorrectly mapped reads or sequence errors in the reads; 2) actual indels detected by resequencing but called as SNPs by the BovineHD BeadChip; and 3) SNPs called by both the resequencing and BovineHD BeadChip but with inconsistent types possibly caused by reads being mapped to wrong positions or by sequence errors either in the reads or in the reference sequences (Table 2 and 3). SNP discovery performance was calculated based on the common SNPs called by more than one pipeline (Figure 3).

**Table 2:** Comparison of heterozygous SNP calls made from BovineHD BeadChip genotype data and the resequencing pipelines.

Pipeline	Consistent	Heterozygous > homozygous	As indel	Inconsistent
BWA + SAMtools	167,758 (93.8%)	10,590 (5.9%)	30	477
CLC Genomics Workbench	175,322 (94.6%)	9,589 (5.2%)	46	438
Mosaik + GigaBayes	158, 564 (97.1%)	4,467 (2.7%)	196	0
SMALT + SAMtools	151,792 (94.1%)	9,097 (5.6%)	31	401

Consistent, the same alleles at the same sites were determined by both the chip and resequencing methods; Heterozygous > homozygous, heterozygotes called on the chip were under-called as homozygote with one identical allele in the resequencing pipelines; As indels, SNP sites on the chip were called as indels in resequencing pipelines; Inconsistent, heterozygous calls with the chip and resequencing pipelines but with different alleles.

**Table 3:** Comparison of homozygous SNP calls made from BovineHD BeadChip genotype data and the resequencing pipelines.

Pipeline	Consistent	Homozygous > heterozygous	As indel	Inconsistent
BWA + SAMtools	253,102 (99.9%)	247 (0.1%)	107	16
CLC Genomics Workbench	254,195 (99.6%)	848 (0.3%)	101	28
Mosaik + GigaBayes	236,261 (97.8%)	3,522 (1.4%)	1,860 (0.8%)	20
SMALT + SAMtools	247,805 (99.8%)	272 (0.1%)	96	19

Consistent, the same alleles at the same sites were determined by both the chip and resequencing methods; Homozygous > heterozygous, homozygous SNPs called on the chip that were over-called as heterozygous in the resequencing pipelines; As indels, SNP sites on the BovineHD BeadChip that were called as indels in resequencing pipelines; Inconsistent, homozygous calls with both the BovineHD BeadChip and the resequencing pipelines but with different alleles.

**Figure 4:**
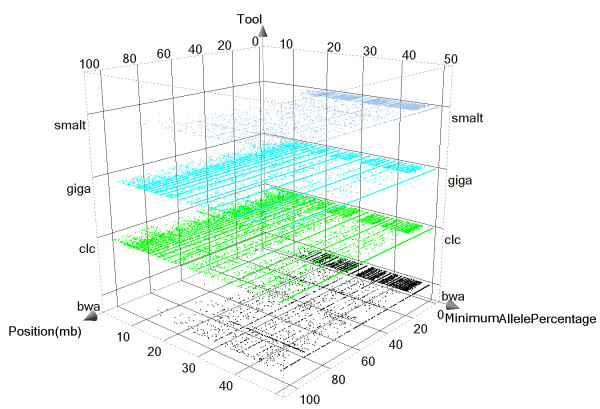
**Heterozygous SNPs called on chromosome 9**. Chromosome 9 contains the largest identical by descent (IBD) region detected in the genome of the sequenced animal. The lower minimum allele percentage of heterozygous calls in the IBD region by both the Mosaik+GigaBayes and CLC pipelines, indicates that even a few false alignments can introduce higher false positive rates (FPRs) compared to the FPRs using the BWA and SMALT pipelines. bwa denotes BWA+SAMtools, clc denotes CLC Genomics Workbench, giga denotes Mosaik+GigaBayes, smalt denotes SMALT+SAMtools.

**Figure 5:**
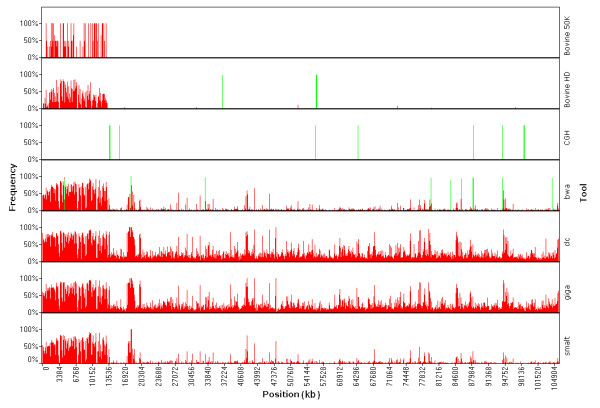
**Interaction of SNP detection and CNVs**. Bases within CNV (green) and heterozygous SNPs (red) regions called on chromosome 9 which contains the largest identical by descent (IBD) region (from 13.2 Mb to 105.6 Mb) in the bovine genome. Some common false positive peaks of heterozygous SNPs in this IBD region detected by all resequencing pipelines overlapped with the CNV regions described in this study and in other literature, indicating a higher FPR for the detection of small size polymorphism inside relatively larger structural variant regions.

**Figure 6:**
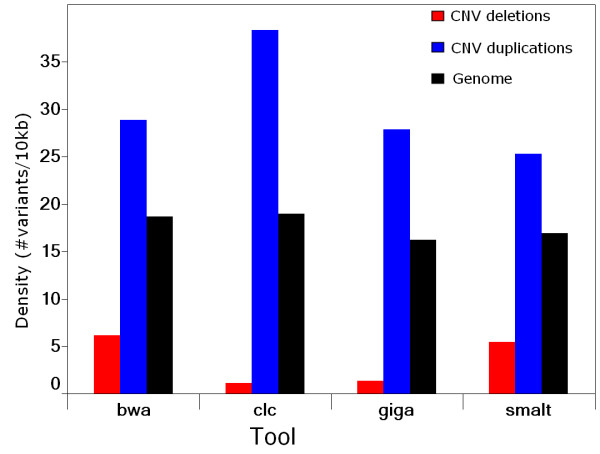
**Densities of small variants detected within CNV regions by different pipelines**. Densities were measured as number of variant per 10 kb averaged across CNV duplication, CNV deletion and the whole genome. smalt, SMALT+SAMtools pipeline; bwa, BWA+SAMtools pipeline; clc, CLC Genomics Workbench; giga, Mosaik+GigaBayes pipeline.

The largest IBD region identified in the sequenced genome with a size of 92.4 Mb (from position 13.2 Mb to 105.6 Mb) was located on chromosome 9. Because a large IBD region can only be due to recent inbreeding, therefore, only few new variants caused by mutations in this IBD region will be observable as heterozygote. Here, the largest IBD region provides an opportunity to estimate the rate of false positive heterozygous calls calculated as the percentage of heterozygous SNPs in this region. Both, the BWA and SMALT pipelines had low false positive rates (FPRs) of 2.6% and 2.7%, respectively. Surprisingly, the CLC and Mosaik pipelines gave much higher FPRs of 19.2% and 19.3%, respectively. Thus, most of the heterozygous sites called by the CLC or Mosaik pipelines in this large IBD region were not identified as variants by either the BWA or the SMALT pipelines. False positive variant calls can be introduced either by sequencing errors or by wrong alignments in which the reads are mapped to improper genomic positions. The SNP detection algorithm of the CLC pipeline is based on Neighbourhood Quality Standard (NQS), whereas Bayesian models are implemented in the other three detection pipelines that were used in the present study. Most of the false SNPs with low-quality discrepancies are likely to be the result of sequencing errors and they can be distinguished by all the algorithms using base quality values and by applying read depth criteria to cover each allele of the SNP. Resolving false SNPs that are likely the result of improper alignments requires a different approach for each of the four pipelines. We used only the reads that were uniquely mapped to reference assemblies in the CLC and Mosaik+GigaBayes pipelines to reduce possible mapping errors, because both these read-mapping and SNP detection algorithms do not utilize mapping quality information [80]. Utilizing mapping quality requires that the aligner considers suboptimal hits and this slows down the alignment process. However, applying a unique mapping strategy does not exclude false alignments as efficiently as filtering with mapping qualities [81]. A recent benchmark test highlighted that filtering using BWA generated mapping qualities with a stringent threshold removed more than 80% of the false alignments at the cost of 1% loss in sensitivity compared with the unique mapping strategy [80,82]. This indicates that the higher number of heterozygous FPR calls with the CLC and Mosaik+GigaBayes pipelines were caused by wrong alignments. Moreover, using only the uniquely mapped reads decreased local read depths and caused an artificial depth bias which affected the detection of both the SNPs and the copy number variations (CNVs). Most false variants caused by wrong alignments should appear as heterozygous rather than homozygous SNPs because it is unlikely that any genomic region will be exclusively covered by false alignments with identical sequence reads. An inspection of the minimum allele percentage (MAP, percentage of reads covering the allele of a heterozygote that has low sequencing depth) of the SNPs called in the largest IBD region showed that most of the false positive (FP) heterozygous SNPs called by GigaBayes and CLC had a MAP that was relatively lower than the MAP of SNPs called by BWA or SMALT, confirming that the FP calls were caused by false alignments (Figure 4). More than 90% of the FP calls were eliminated by filtering with a coverage > 2 and a minimum variant frequency > 35% for the heterozygous SNPs identified by CLC. While adding this extra filtering step after variation detection can reduce FPs caused by false alignments, it is at the cost of a significant loss in sensitivity, especially when the sequencing depth was moderate. These results show that it is essential to utilize alignment mapping quality to reduce the FPR caused by the wrong alignment of short NGS sequences with low to medium sequencing depth. The several FP heterozygote peaks that were common to all the resequencing pipelines used are most likely to result from assembly errors or structure variants rather than from random errors from the pipelines. Some of the highest common FP heterozygous peaks were located in the CNV regions detected by both CNV-seq and the CGH array (Figure 5), indicating a decrease in SNP detection accuracy within the CNV regions. The density of the small variants, the SNPs and the indels < 12 bp long, that were detected inside the CNVs by the four pipelines showed a strong dependence upon whether the CNV was a duplication or a deletion. The density inside CNV duplications was much higher than the density that was observed at the whole genomic level; for CNV deletions it was the opposite. The density of the small variants detected by the pipelines that take advantage of mapping quality (BWA and SMALT) within CNV duplications was about 5 times higher than their density within deletions. For the pipelines that do not use mapping quality, the ratio of their densities was even higher (Figure 6). Reads that were derived from CNV duplications in the target genome and that mapped onto the corresponding reference paralogues resulted in the prediction of more false small variants because the copies in CNV regions of these reference sequences do not always have identical sequences. In contrast, the reads that were derived from CNV deletions in the target genome and that were mapped to copies in the reference paralogues either had reduced mapping quality or were rejected when a unique mapping criterion was applied. These results ultimately lead to lower sequence coverage which, in turn, causes an underestimation of the predicted variant density in CNV deletions. The algorithms that apply mapping quality reduced this kind of bias significantly; however, the bias was not completely avoided.

**Figure 7:**
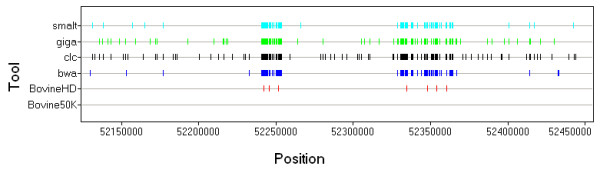
**False heterozygous SNPs called within identical by descent (IBD) regions**. Some false heterozygous SNPs called inside the IBD region on chromosome 9 by both the BovineHD BeadChip and resequencing pipelines were clustered close to each other, suggesting that they were caused by some systematic errors instead of random errors.

In addition, there were 15 heterozygous SNPs called by the BovineHD BeadChip in this largest IBD region which was more than the expected number according to its calculated error rate. Seven out of the 15 SNPs were confirmed to be heterozygous with identical alleles called by all four resequencing pipelines; one of the SNPs was confirmed by three of the resequencing pipelines and another one was confirmed by two of the resequencing pipelines. These nine heterozygous SNPs called by both the SNP chip and resequencing pipelines, appear to have been caused by some systematic error. When the nine SNPs were checked manually, six of them were found to be located close to each other in a 119 kb block that gave a much higher density of heterozygous calls than in the flanking areas; an abnormally high depth of mapped reads were also found upstream to this block, strongly suggesting either an assembly error or some structural genomic event in this region. Two of the remaining three SNPs were also located in a region that had a relatively higher density of heterozygous calls than its flanking areas, indicating that they were also caused by either an assembly or mapping error (Figure 7). The last heterozygous site that was called by both the BovineHD BeadChip and the four resequencing pipelines with identical alleles may be a new mutation that occurred in the IBD region during the last three generations and that was inherited following the common ancestor. In this study, the estimated BovineHD SNP chip heterozygous detection rate was 0.09 (Additional file 4), giving us a deduced mutation rate of 1/0.09/2/3/90M = 2e-8 (See also additional file 4), similar to the magnitude seen in other studies [83].

### Structural Variation detection

To identify the whole spectrum of genetic variation, the integration of different methods is a necessary task. In this section, the analysis and integration of different structural variant detection methods is described.

#### Intra-read indels detection

Although present at lower rates than SNPs, small insertions and deletions (indels) represent a functionally important type of genomic variation [20,84-87]. The false positive rate of indel detection is generally higher than for SNPs because reads bearing indels often align with multiple mismatches to the reference sequence rather than leave a gap, and because indels frequently cannot be uniquely mapped onto the reference, for example, when the indel is in a homopolymer repeat. Therefore, to minimize false positives, the intra-read indels were here identified as those that were called by both the Dindel and SAMtools variant calling programs (details in Methods) [54,57]. We identified 197,895 intra-read indels, of which 93,210 were deletions of up to 12 bases and 104,686 were insertions of up to 10 bases at an average genome density of 7 indels per 100 kb. This indel size distribution is close to a normal distribution (Additional file 5).

**Figure 8:**
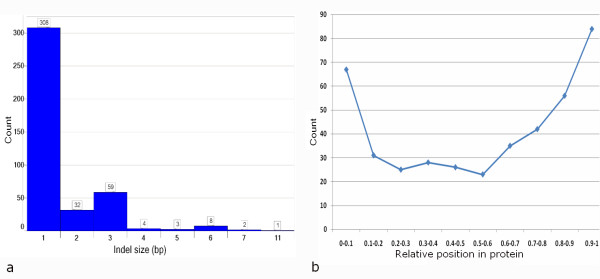
**Indels in coding regions of the genome**. (a) Indel size distribution in the coding regions. The indels called by all methods demonstrate strongest purifying selection against frameshifts (non 3 n) indels in coding regions. (b) Relative protein location of coding indels. The relative position of the indel in the protein was calculated by dividing the position of the indel by the total protein length.

We found 417 indels (0.21%) in the coding sequences of 368 genes and observed that these coding indels were enriched for sizes that are multiples of three (3 n). The enrichment of 3 n coding indels compared to 3 n genome-wide indels may be explained by purifying selection against frameshifts in coding regions (Figure 8a). A significant under-representation of indels in coding regions was also detected. Whereas the genome-wide SNP:indel ratio is 15:1, the SNP:indel ratio for coding regions is 67:1, as would be expected because of the more deleterious effect of indels in protein-coding regions [87].

Next, we investigated the location of the coding indels in the respective proteins and found that the indels were enriched near the N and C-termini of the proteins with a slightly higher frequency at the C-termini (Figure 8b). This distribution of coding indels is consistent with observations from previous studies [43,87], and can be explained by the fact that a C-terminal indel would have a lower likelihood of affecting protein function because the major part of the protein would already have been translated before the indel is encountered. A coding indel at the N-terminus may also be less critical because a possible alternative downstream start codon may be present. Thus, indels at the N- and C-termini of proteins may be less functionally constrained than indels at other protein locations. We also analyzed the possible functional importance of coding indels in genes that are known to be involved in disease (OMIM database [70]). Of the 24 indels found in 20 disease genes, 18 of the indels were not multiples of 3 n, which would induce frame shifts in these genes (Additional file 6). Further, in the present study, a significant genome-wide correlation of SNP-indel density was discovered (Additional file 7, Pearson correlation R^2 ^= 0.4, p < 0.05). This correlation was previously reported in human and other primate genome studies and seems to be a general phenomenon [88-91].

Because, in this study, an indel was called by at least a 4× sequence coverage, we accept that this prevents at least 10% of the genome being reachable to indel detection (Figure 1). Furthermore, indels are seriously underestimated in regions of local repetitive and homopolymeric sequences.

#### Indels, inversions and translocations found by split-read and mate-pair approaches

**Figure 9:**
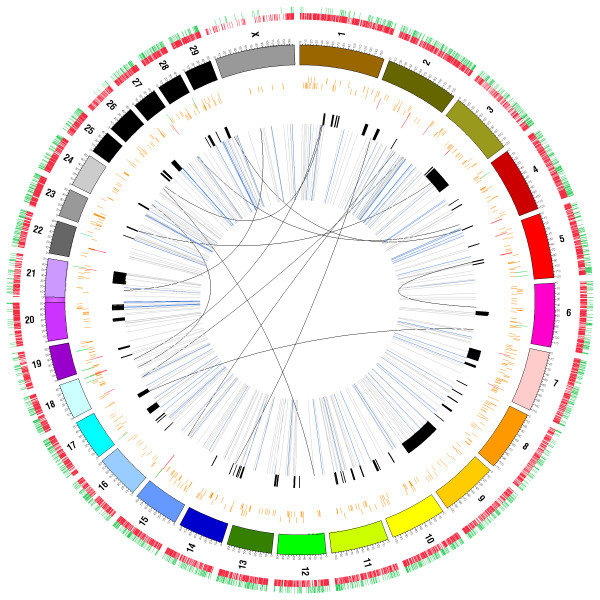
**Genomic distribution of structural variation**. The innermost circle shows inversions (grey line) and intra and inter-chromosomal translocations (blue and black lines, respectively). Black bars represent IBD regions identified in the bull. The next circle shows a histogram representing deletions (red) and duplications (green) found by at least two of the CNV platforms; CNVs called by only one platform are in orange. The circle outside the ring of solid coloured chromosomes represents the insertions (green) and deletions (red) found by the mate-pair (BreakDancer) and split-pair (Pindel) analyses.

Although small intra-read indels can be detected by SAMtools and Dindel variant callers, to detect larger structural variants other tools have to be used. Here we applied Pindel that uses a split-read approach to examine unmapped reads spanning breakpoints, and BreakDancer that examines discordantly mapped read pairs having improper orientation relationships or span sizes [58,59]. Both tools detect insertions and deletions as well as inversions and translocations (Figure 9).

Using the BreakDancer tool we detected a total of 8,768 structural variants (SVs). Of these, 6,156 were deletions (ranging in size from 47 bp to 127 kb), 2,125 were insertions (241 bp to 397 bp), 412 were inversions (3 bp to 89 kb), and 120 were intra- and 18 inter-chromosomal translocations. In total, the SVs overlapped 2,529 genes. Inversions, intra- and inter-chromosomal translocations were later filtered and kept only if their positions could be located within the same chromosomes in both cow assemblies (Btau4.0 and UMD3.1). This reduced the total number of these variants by 28%, with inversions decreasing from 412 to 297, intra-chromosomal translocations from 120 to 86, and inter-chromosomal translocations from 18 to 13 (Additional file 8). Using the Pindel split-read approach, 1,416 SVs, including 1,332 deletions (13 bp to 855 kb), 79 insertions (15 bp to 31 bp) and 5 inversions (7 bp to 28 bp) (Additional file 9) were detected. In total, these SVs overlap nine genes.

**Figure 10:**
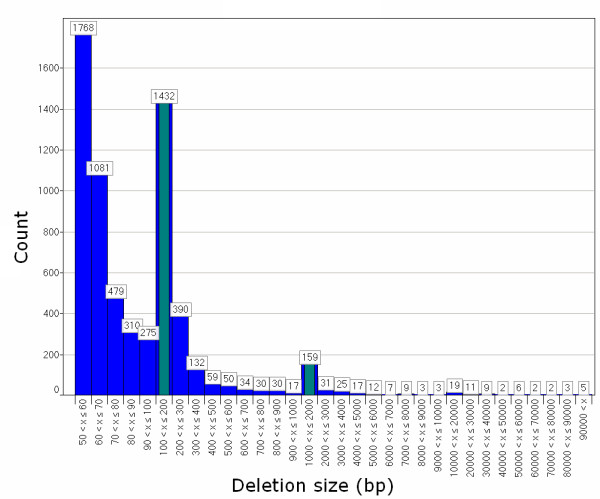
**Size distribution of deletions detected by BreakDancer and Pindel**. With increasing deletion size, the frequency decreases exponentially. The exceptions are for the bars (green) that correspond to SINE and LINE elements.

Pindel and BreakDancer are largely complementary in that they identify SVs with different signatures. Pindel produces base-pair resolution for SV boundaries and consequently can detect significantly smaller SVs than BreakDancer. Both tools identified more deletions than insertions, probably due to a bias against detection of insertions longer than our paired-end library span size. Nevertheless, we cannot rule out the possibility that deletions are more common, as hypothesized recently [88]. We also noted that the number of SVs decreased rapidly with increasing size, except for two peaks corresponding to short and long interspersed nuclear elements (SINEs and LINEs) around 200 bp and 2000 bp, respectively (Figure 10). This result is consistent with previous studies that reported the presence and distribution of these repeat elements [24,34,36,37].

### Copy number variation

Three platforms were used for the detection of copy number variation (CNV); by sequencing, and by SNP and CGH arrays.

#### Array based variation

A high density custom Nimblegen comparative genomic hybridization (CGH) array with 6.3 M probes and the Illumina BovineHD BeadChip with 770 K probes were used to identify relative copy number variants in the bull sequenced in this study. The CGH array data was available from a previous study in which the bull was hybridized to two other animals using a dye swap design [29]. From the CGH array set we called 196 CNVs (6.11 Mb) that had an average length of 31.2 kb and ranged in size from 1.4 to 595.7 kb (Additional file 10). We used the BovineHD BeadChip array to detect CNVs in our bull when compared with a reference population of Holsteins that were also tested in-house. Details of the calling procedure are described in the Methods section. Briefly, we used the PennCNV tool to detect the CNVs and after visual plot inspection we identified 30 CNVs (2.57 Mb) with an average length of 85.8 kb, that ranged in size from 8 to 555.3 kb (Additional file 11). This result reflects the lower resolution of the BovineHD BeadChip compared to CGH array which makes it less efficient in detecting short CNVs.

#### Sequence based variation

We also used a sequence based approach, called CNV-seq [60], in which the sequencing depth of coverage of our bull was compared with the sequencing depth of coverage of another bull sequenced in another study [46]. Assuming a uniform sequencing process, the number of reads that map to a particular region should be Poisson distributed and proportional to the copy number of the reads. Despite this theoretical assumption, this and previous studies have reported a sequencing Poisson overdispersion [34,89]. This overdispersion is probably due to the known sequencing bias of the second generation sequencing technologies that causes certain regions of the genome to be over or under sampled; GC-rich regions and homopolymeric tracts are the best known causes of this bias [63-65]. To minimize the bias, the detection of CNVs in our bull was done by comparing it to another bull sequenced on the same sequencing platform with the same read length and the same aligner. Here, based on the depth of coverage signature, we were able to detect 520 autosomal CNVs (3.63 Mb) with an average length of 6.9 kb ranging in size from 3.2 to 129.9 kb (Additional file 12).

#### Cross-platform CNV comparison

**Figure 11:**
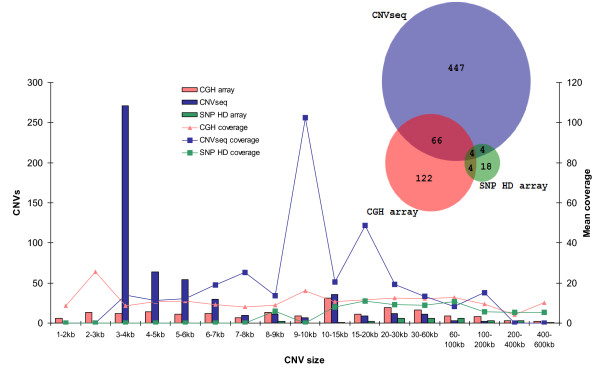
**Comparison between different platforms**. The Venn diagram depicts the number of CNVs that overlap among the three platforms; CGH array (red), CNV-seq (blue), and SNP HD array (green). The bar plot depicts CNV size distribution for each platform in each of the size ranges; the line plot represents the mean coverage of CNVs found in each size range.

Notwithstanding the large differences in the numbers of CNVs identified by the three platforms, the CNV genome coverage was similar; 0.10% for the BovineHD BeadChip, 0.14% for CNV-seq, and 0.24% for the CGH array. To compare the CNV datasets from the three platforms, a CNV was described as overlapping another CNV if there was an overlap of at least 1 bp. About 14% of the CNVs detected by CNV-seq, nearly 23% of the CNVs detected by the CGH array, and 23% of the CNVs detected by BovineHD BeadChip overlapped CNVs detected by at least one of the other platforms. Figure 11 shows an overall comparison between the different platforms.

The apparently low overlap of CNVs detected by the different platforms might have several different causes. First, each platform has different advantages and disadvantages in terms of resolution and size distribution for confident CNV calls (Figure 11). Second, coverage bias also affects the CNVs called; the coverage range at which CNV-seq can detect CNV is much higher than for the array platforms because of microarray signal saturation at high copy levels (Figure 11). Array coverage putatively correlates with copy number status of the region. Third, the CNVs discovered by each platform are relative to the animals that are probed against. Hence, while for the CGH array two samples were hybridized against our bull, a different population was used as the reference population for calling CNVs using the SNP platform and again, for CNV-seq, a different animal was tested against our bull. Therefore, some of the relative CNVs that we found may not have originated from our sequenced bull but may have come from the animals that we tested the CNVs against.

#### CNV validation by quantitative PCR

To verify whether any of the three reasons listed above are valid, a subset of 28 CNVs was selected for testing using quantitative RT-PCR. Five CNVs unique to each of the platforms, nine CNVs found by two platforms and four CNVs detected in all platforms formed the subset. We used a DNA sample of the bull sequenced here, samples from the two animals hybridized to the bull in the CGH array experiment and DNA from four of the animals used as our reference population in the BovineHD BeadChip array. The primers did not work in six of the 28 possible RT-PCR experiments. Of the remaining 22 CNVs, 19 (86%) worked and gave positive results, meaning that a CNV was detected in at least one of the animals tested (Additional file 13). Despite the fact that the CNVs tested by RT-PCR were a small subset of the total number of CNVs found by all platforms, the results gave us an estimate of the real reasons behind the lack of CNV overlap. Thus, for the CNVs tested by RT-PCR that were unique to the CGH and SNP arrays, the majority were detected in other animals and not in our bull. The CNVs that were detected in our bull were in regions of low sequence coverage which can affect the calling procedure of the CNV-seq method (Figure 11, t-test p < 0.05 for CNV-seq which can detect significant CNVs with higher coverage than the array based methods). The RT-PCR results provided confidence that, for the genome-wide scan, the false positive rate was low in CNV regions; however, the penalty was a relatively high false negative rate when a single method was used alone.

#### Functional analysis of CNVs

To test for the putative functional significance of our CNVs, we restricted our analysis to the CNVs found by at least two platforms; this should reduce the false positives and the risk that the CNVs are not found in our animal but only in others. From 46 CNV regions that were detected using at least two platforms, 27 (59%) were overlapped by 84 genes giving a gene enrichment of 2.6 fold in comparison with putative CNVs distributed at random locations. Similar to our results for the SNPs, this gene enrichment is biased for functions related to olfactory transduction, and signalling pathways including immune response (Additional file 14) and is consistent with previous bovine and human CNV studies [19-22,27-30,92]. This commonplace enrichment involves the genes that encode proteins that interface with the external environment and its selective pressures (natural or artificial). These pressures results in differential adaptation to different metabolic and immune conditions (particularly important in the rumen micro-biome and mucosal surfaces), to herd environment, and to the artificial genetic selection that has significantly impacted bovine genome evolution. However, to support an argument for their potential evolutionary contributions to cattle domestication, breed formation and adaptation, the genomic variations reported here need to be queried in a larger sample size and with breeds other than Holstein. Comparisons to other farm animal species could also provide additional insights into the evolutionary mechanisms of genomic variation in livestock research. CNV regions are also enriched in segmental duplications which are known substrates of copy number formation [21]. We found no enrichment of CNVs at evolutionary breakpoints when cattle-specific and artiodactyl-specific evolutionary breakpoints [93] were examined, in agreement with a previous study [92]. It is interesting that our most stringent set of 46 CNVs included 35 (76%) CNVs that overlapped with common (frequency > 2.5%) cattle CNVs reported in previous studies [27-30] (Additional file 15).

The high-confidence CNV dataset for the individual bull sequenced here contains CNVs that were identified by at least two platforms. This dataset could be used as a reference control sample in future array CGH experiments. It would help alleviate doubts of whether a particular CNV is a gain or a loss in the investigated sample because the absolute copy number status of the reference animal is known. This strategy is similar to one that is already used in human studies [91,94].

### The detected genetic variation present in the bull's genome

After calling both the sequence and structural variations, we generated an overview of the genetic variation found in the Holstein-Friesian bull sequenced here. For the overview we used the SNPs called by all four methods (Mosaik+GigaBayes, CLC Genomics Workbench, SMALT+SAMtools and BWA+SAMtools), the intra-read indels called by SAMtools and Dindel, indels, inversions and translocations predicted by Pindel and BreakDancer, and finally the CNVs called by at least two of the platforms (CNV-seq, CGH and SNP HD arrays). While there were 2,859,650 bases of sequence variation, the structurally variable part of the genome comprised 11,672,807 bases, a finding supported by previous studies claiming that structural variation surmounts sequence variation as the main form of genetic variability, measured as the number of basepairs affected, in an individual genome [20,95]. A summary of the different analyses that were performed is shown in Table 4.

**Table 4:** Genetic variation detected by different methods (size in base pairs).

Method	Type	Number	**Min**.	Median	Max	Length
Mosaik+GigaBayes+CLC+BWA+SMALT+SAMtools	SNP	2,859,650				2,859,650
SAMtools+Dindel	indel	197,895	1	1	12	349,248
BreakDancer (mate-pair)	indel, inv, transl	8,596	42	122	127,536	6,286,578
Pindel (split-read)	indel, inv	1,416	13	22	855,018	1,147,264
CNV-seq+Nimblegen6.3M+Illumina770 k	CNV	46	3,170	25,812	595,739	3,889,717

**Total**		**3,067,603**	**1**		**855,018**	**14,532,457**

inv denotes inversion, transl denotes translocation.

## Conclusions

This study presents an in-depth analysis of the genomic variation in the genome of a single bull at a comprehensive scale and resolution for *Bos taurus *species. Genetic variations comprising SNPs, indels and large size structural variants like CNVs were explored using several complementary technological platforms and analysis software. We demonstrated that all the platforms were powerful tools for the identification of sequence and structural variations and that the various technologies complemented each other. For instance, CNV discovery by sequencing enabled the efficient detection of small, highly variable CNV regions, while the CGH and SNP platforms were better at detecting larger CNVs with smaller copy number differences (Figure 11) [95]. The large number of platform-specific CNVs and smaller number of false positives (as shown by RT-PCR validation) indicated how all these platforms complement each other in CNV discovery. Here, we propose that a genome-wide picture of false positive and false negative rates can be improved using sequencing trios.

For SNP detection with strict alignment criteria, the inclusion or exclusion of non-uniquely mapped reads did not significantly change the accuracy or rate of detection; however, using only uniquely mapped reads may reduce the number of detectable small insertions and deletions. The SNP detection algorithms implemented in the Mosaik+GigaBayes and CLC Genomics Workbench pipelines are Bayesian and Neighbourhood Quality Standard respectively, but neither of them includes alignment mapping quality in the model. This leads to a relative higher false positive rate in heterozygous variants compared to the other Bayesian based approaches, the BWA+SAMtools and SMALT+SAMtools pipelines, with mapping quality implemented. Proper filter setting with minimum allele percentage for heterozygous sites was essential to minimize the false positive calls in the detection pipelines without mapping quality; however, this filtering causes a serious loss in detection sensitivity. This study also clearly documents that applying more than one algorithm/tool to call common variants increased detection accuracy at the expense of sensitivity. If multiple analyses using various algorithms/tools are hard to achieve, the BWA+SAMtools pipeline could be considered to be a good balanced choice. The detection of small variants located inside large structural variants like CNV regions can be problematic for all algorithms, mainly because short reads from different but highly similar structural sequence elements inevitably map and cluster together on the reference genome. Genetic variants inside and near to those structural components should always be critically evaluated.

In summary, we found that structural variation surpassed sequence variation as the main component of genomic variability. This emphasizes the need to consider all types of variants when fine-mapping causal variants within trait-associated intervals. Furthermore, our results suggest that, at the level of resolution and sequencing coverage found in the present study, an ensemble of platforms and tools can be used to maximize the detection of SV; however, the false positive rates should be controlled by applying threshold settings and performing subset validation experiments.

The methodology used in this study has a number of limitations that should be addressed in future work. One limitation relates to calling variants embedded in repetitive regions which requires longer sequencing read lengths for complete characterization [96,97]. *De novo *local assembly could also help to resolve and validate the breakpoints of structural variants [98,99], but the present sequencing coverage prevented us from investigating this approach. Using paired-end sequencing of additional libraries of different sizes will also help resolve complex variants. With sequencing costs decreasing rapidly, simply increasing the depth of sequencing and using different mapping procedures would increase the confidence and accuracy of the results [100].

It could be argued that the relatively low read-depth achieved here compared to other personal genome studies [34-39] may affect the accuracy of variant classification (whether by under- or overcalling) [101]. However, we clearly demonstrated that platform integration can mitigate such problems for structural variation detection, especially if the interest is in gaining a broader understanding of the genomic characteristics of a breed or population group and is not primarily focussed on understanding the detailed genomic architecture of a specific animal. Thus, we suggest that it would be more prudent to sequence many individuals with lower depth rather than a limited number of individuals with high depth. The integrative methodology and resources generated for this study may be used as a template for future genome sequencing studies on larger data sets.

## Methods

### Sequencing

Genomic DNA from a Holstein-Friesian bull was extracted and purified from blood according to standard protocols and as previously described [102]. Sample preparation, cluster generation and sequencing were performed according to the manufacture's protocols with minor modifications (Illumina paired-end cluster generation kit GA II v1, 36-cycle sequencing kit v1.2 and v1.3). Briefly, two paired-end libraries were prepared and sequenced using a Genome Analyzer II (Illumina, San Diego, California, USA). Genomic DNA was sheared by nebulization, ligated with Illumina's PE adaptors, and fragments approximately 300 and 500 bases in length were gel purified followed by PCR amplification and column purification. Purity and yield were checked using the 2100 Bioanalyzer (Agilent Technologies, Santa Clara, California, USA) and yields were additionally measured using the QuBit (Invitrogen, Carlsbad, California, USA). To extract intensity measurements for each cluster and sequencing cycle, image analysis was performed with the Illumina Firecrest program as implemented in Illumina's pipeline version 1.3. Illumina's Bustard program was used for base calling on the extracted intensities; with purity filtering was applied to discriminate between good and bad reads. The quality score for each base was used as an indicator of base call uncertainty.

### Reference genome assemblies

We used the Btau4.0 [103] assembly from The Bovine Genome Sequencing and Analysis Consortium and the UMD3.1 assembly [104] from the Center for Bioinformatics and Computational Biology at the University of Maryland as two independent references for mapping and assembling the whole genome shotgun Illumina reads [2,67].

### SNP detection by sequencing

Short read alignment, consensus assembly and variant calling were performed using the BWA v0.5.8 and Mosaik v1.0 software packages, SAMtools v0.1.12a, GigaBayes v0.4.1, and the CLC Genomics Workbench v4. We used the default parameters for the BWA alignment. The SAMtools pileup command was used for variant detection in BWA pipeline with default parameters but for filtering, a minimal mapping quality of 20 was used. Options -n 8, -j 50, and -i 650 were used for the SMALT alignments. The SAMtools pileup command was used for variant detection in SMALT pipelines with default parameters but for filtering a minimal mapping quality of 20 was used. Parameters -hs 15 -mm 2 -a all -m unique -mhp 100 -act 20 were applied for Mosaik mapping in the Giga pipeline. GigaBayes was run in the Giga pipeline for variant detection with the following parameters: --indel --sample single --ploidy diploid (haploid for the sex chromosomes) --CRL 2 --CRU 60 (30 for the sex chromosomes) --QRL 20 --PSL 0.9 --anchor --O 3. CLC Genomics Workbench was used for mapping reads with parameters set as: -p fb se 150 650. The following CLC parameters were applied for variant detection: Maximum coverage = 60 (30 for the sex chromosomes); Maximum expected variations (ploidy) = 2 (1 for the sex chromosomes); Maximum gap and mismatch count = 2; Minimum average quality = 15; Minimum central quality = 20; Minimum coverage = 2; Minimum paired coverage = 0; required variant count threshold = 1; and the Sufficient variant count threshold = 2.

### Structural variation detection by sequencing

Structural variants (excluding CNVs) were detected by using the BWA mapping result on the UMD3.1 assembly. Intra-read indels were detected by the intersection of Dindel v1.0 and SAMtools mpileup variant callers with default parameters. After merging the two indel sets, post-filtering was applied. Indels were kept if: (1) the non-reference allele was covered by at least one read for each strand; (2) the minimum base quality was 20; (3) the coefficient for downgrading mapping quality for reads containing excessive mismatches was 50; (4) the minimum read depth was 4; (5) the maximum read depth was 30, and (6) the indels did not overlap Ns in the assembly. For indels and inversions found by split-read, Pindel v0.2.0 was used with default parameters. Post-filtering was applied to remove structural variants not seen in both strands and variants having read depth < 4. For indels, inversions and translocations found by mate-pair approach, BreakDancer v1.1 was used with default parameters, except that the minimum alternative mapping quality was set to 20, the minimum number of read pairs required to establish a connection was 4, and the maximum threshold of haploid sequence coverage for regions to be ignored was 50.

For CNV detection, CNV-seq was used with reads mapped to assembly Btau4.0. Btau4.0 was chosen because our previous array CGH study was designed for this assembly and to compare the CNV detection platforms, the reads all had to be mapped to the same assembly. Maq v0.7.1 [80] was used to map the reads from our sequenced bull and another bull sequenced elsewhere [46], while CNV-seq was used to compare the normalized read-depth differences between the two animals in a sliding windows across the autosomes. A CNV was kept if the |log2ratio| of the counts of reads per sliding window was bigger than 1, and if the CNVs were called by at least 5 consecutive windows.

### Array based SNP chip genotyping

The sequenced animal was genotyped with BovineHD and BovineSNP50 BeadChips (Illumina, San Diego, California, USA). To accurately detect chromosomal positions of the SNP sites, Tera-Blast (Timelogic, USA) was used for mapping the flanking SNP sequences against both the UMD3.1 and Btau4.0 genome assemblies [80,81]. Only those SNPs with unique perfect hits were used. GenomeStudio v1.7.4 software (Illumina, San Diego, California, USA) was used to detect the SNPs for both chips and both assemblies. The BovineHD BeadChip was also used to detect CNVs in Btau4.0. Briefly, signal intensity (log R ratio, LRR) and allelic intensity (B allele frequency, BAF) values were extracted, and used by PennCNV to detect CNVs in our bull by comparing with a reference population of 138 other Holstein bulls that have been genotyped (data not shown) [61]. Only high quality samples that had a call rate > 99.9%, standard deviation of log R ratio (LRR) below 0.3, BAF < 0.01 and wave adjusted values < 0.05 were used. A CNV was detected if 5 consecutive SNPs showed the same CNV pattern and if this pattern was confirmed by visually inspection of signal intensity plots.

### Array CGH

The array CGH experiment has been described elsewhere [29]. Briefly, a custom 6.3 million probe array was produced by Nimblegen (Roche Nimblegen, Madison, Wisconsin, USA) to cover the Btau4.0 genome assembly at 301 bp median probe spacing. Twenty-one animals were tested for CNVs and the data from three of them (the bull sequenced for the present study and the two animals that were hybridized with it) were used for this study.

### Functional analysis software

Variant effects were analysed based on both the UMD3.1 and Btau4 genome annotations [67,105] using customized perl scripts. The functional effects of non-synonymous SNPs on the coded protein were predicted by SIFT and PolyPhen [71,72]. Gene set enrichment analysis was performed with the DAVID bioinformatics resources v6.7 (with medium stringency) [106]. BEDtools and liftover utilities were used for data extraction, variant manipulation and overlap of genome annotations [107,108].

### Quantitative Real Time PCR

Validation of the subset of CNV regions discovered by sequencing, CGH and SNP arrays was performed as previously described [29]. Briefly, assays were run on an Applied Biosystems 7900 HT Sequence Detection System and downstream analysis was done with the SDS 2.2 software following the guidelines of the manufacturer (Applied Biosystems). Primers and probes (Universal ProbeLibrary Probes, Roche Applied Science) were designed using the ProbeFinder software from Roche Applied Science (Additional file 13). In total, seven animal samples were assayed (samples from our sequenced bull, 2 animals from the array CGH [29] and 4 from the SNP array were used) using the sequenced bull as the reference sample. For each target, the relative quantification analysis with the reference sample was done to calculate estimated copy numbers for each sample.

### Data access

All purity filtered read data from the sequenced bull is available at EBI sequence read archive, accession number: ERP000712.

## Abbreviations

BAF: B Allele Frequency; CGH: Comparative Genomic Hybridization; CNV: Copy Number Variation; FP: False Positive; FPR: False Positive Rate; GWAS: Genome-Wide Association Study; IBD: Identity by Descent; Indel: Insertion/deletion; LRR: log R ratio; MAP: minimum allele percentage; NQS: Neighbourhood Quality Standard; PCR: Polymerase Chain Reaction; ROH: Run of Homozygous; RT-PCR: Real Time PCR; SNP: Single Nucleotide Polymorphism; SV: Structural Variation; UMD3.1: Bos Taurus 3.1 assembly by the Center for Bioinformatics and Computational Biology at the University of Maryland.

## Competing interests

The authors declare that they have no competing interests.

## Authors' contributions

BZ, JF, FP, and CB designed the project. BZ and JF performed the data analysis and drafted the manuscript. BT planned the RT-PCR validation experiments. JH planned the resequencing and performed the basic sequence analysis. CB was the principal investigator of the project. All the authors have contributed to writing this manuscript and have read and approved the contents of the final submitted version.

## Supplementary Material

Additional file 1 **Summary of short sequence read mapping methods and results**. The data is listed in a table that displays different mapping methods and corresponding results. Click here for file

Additional file 2 **Read depth plot**. The read depth mapped on assembly Btau4.0 by Mosaik mapping tool. Click here for file

Additional file 3 **Graph of pedigree**. The pedigree of the sequenced bull traced back five generations. Click here for file

Additional file 4 **Calculating description**. A description of the methods used to calculate the BovineHD BeadChip detection rate and the mutation rate of the sequenced animal. Click here for file

Additional file 5 **Indel sizes**. The intra-read indel size distribution detected by Dindel and SAMtools mpileup. Click here for file

Additional file 6 **Disease gene list**. The genes affected by coding indels that are known to be involved in disease (OMIM database). Click here for file

Additional file 7 **SNP-indel density correlation**. The correlation of SNP-indel densities in chromosome 1 at 20 kb intervals. Click here for file

Additional file 8 **Structural variant list**. The structural variants detected by the BreakDancer tool (UMD3.1 coordinates). Click here for file

Additional file 9 **Structural variant list**. The structural variants detected by the Pindel tool (UMD3.1 coordinates). Click here for file

Additional file 10 **CGH CNVs**. The CNV data from the array CGH (Btau4.0 coordinates). Click here for file

Additional file 11 **BovineHD BeadChip CNVs**. The CNV data from the BovineHD BeadChip (Btau4.0 coordinates). Click here for file

Additional file 12 **CNV-seq CNVs**. The CNV data from read-depth of sequencing using CNV-seq (Btau4.0 coordinates). Click here for file

Additional file 13 **Validation of CNVs**. CNV validation result using RT-PCR. Click here for file

Additional file 14 **Result of gene enrichment analysis**. Gene and pathway enrichment for CNVs found using at least two platforms (DAVID database). Click here for file

Additional file 15 **Reported CNVs**. Common (frequency > 2.5%) cattle CNVs that overlap with CNVs from previous studies. Click here for file
