# Supplementary material for: Global assessment of genomic variation in cattle by genome resequencing and high-throughput genotyping
Source: BMC Genomics. 2011 Nov 14;12:557. doi: 10.1186/1471-2164-12-557 (PMC3248099; doi:10.1186/1471-2164-12-557)
Supplement: Additional file 1 — Summary of short sequence read mapping methods and results. The data is listed in a table that displays different mapping methods and corresponding results. [file 1471-2164-12-557-S1.PDF]

Summary of mapping methods and results

**Tools**	**Alignment**	**Reference**	**Coverage**	**Mean depth**	**Note**
Bwa	Gapped	UMD3	98.3%	14.8 X	
CLCBio	Gapped	UMD3	87.0%	9.1 X	Uniquely mapped reads
Smalt	Gapped	UMD3	97.2%	13.2 X	
Mosaik	Gapped	UMD3	82.8%	7.1 X	Uniquely mapped reads
Mosaik	Gapped	Btau4	75.3%	6.5 X	Uniquely mapped reads
