# Supplementary figures and images for: Global assessment of genomic variation in cattle by genome resequencing and high-throughput genotyping
Source: BMC Genomics. 2011 Nov 14;12:557. doi: 10.1186/1471-2164-12-557 (PMC3248099; doi:10.1186/1471-2164-12-557)

# Depth on assembly bosTau4.0 by Mosaik

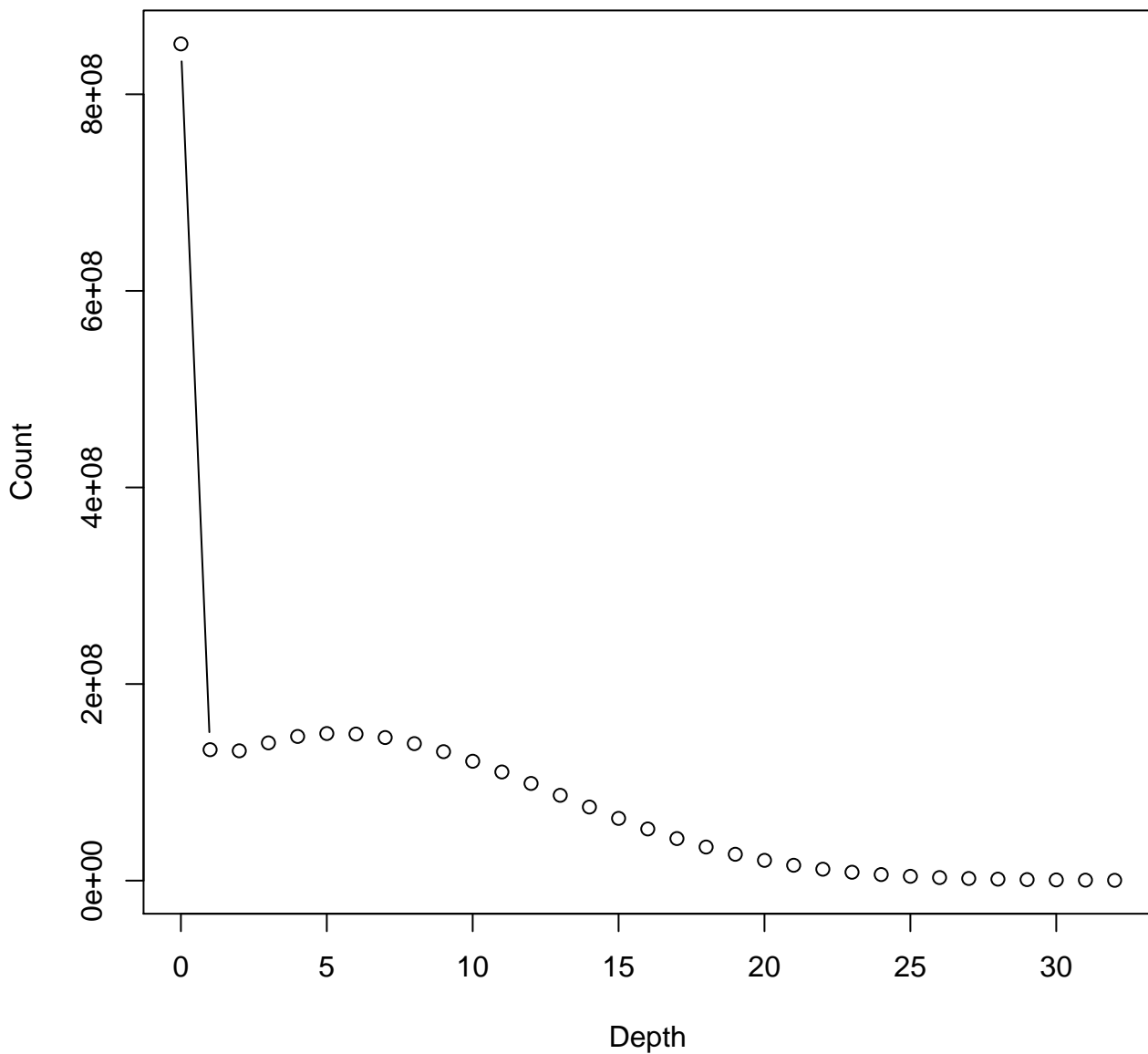

Supplement: Additional file 2 — Read depth plot. The read depth mapped on assembly Btau4.0 by Mosaik mapping tool. [file 1471-2164-12-557-S2.PDF]

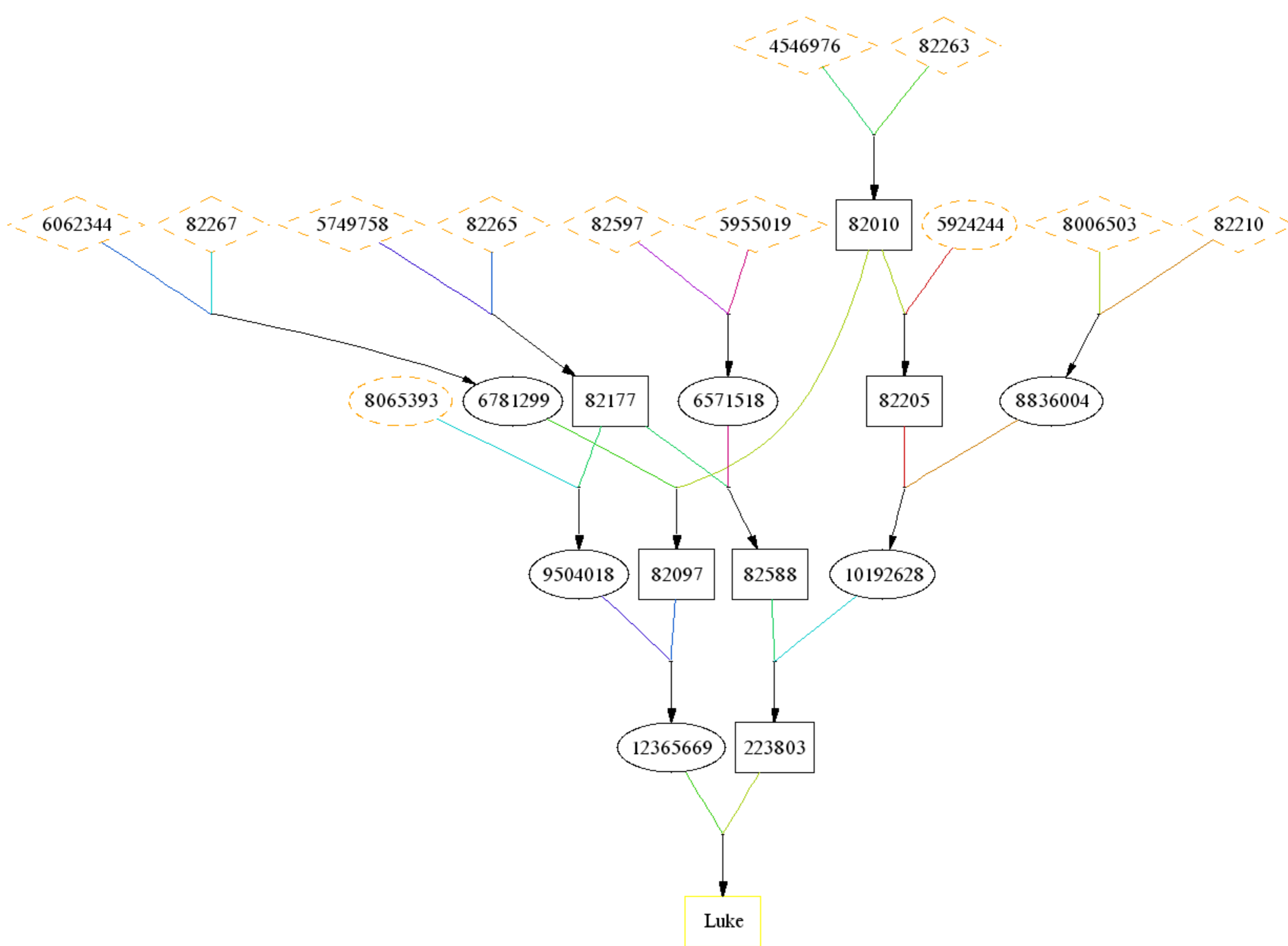

Supplement: Additional file 3 — Graph of pedigree. The pedigree of the sequenced bull traced back five generations. [file 1471-2164-12-557-S3.PDF]

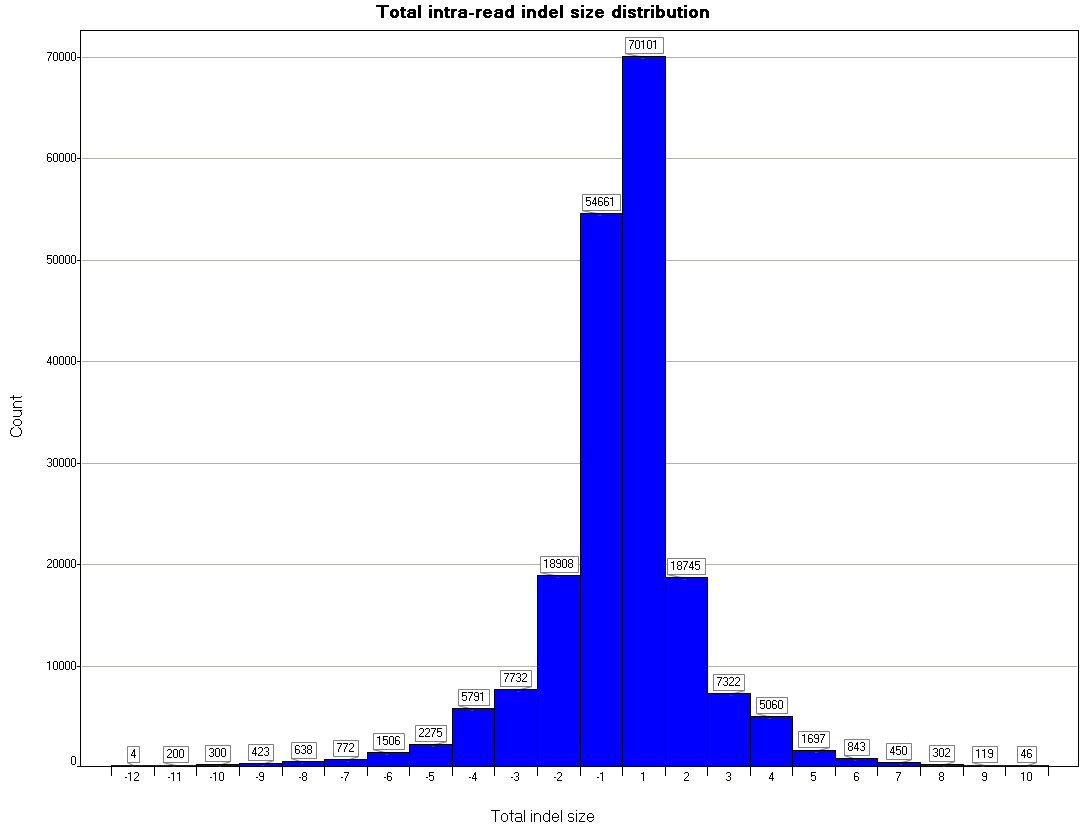

Supplement: Additional file 5 — Indel sizes. The intra-read indel size distribution detected by Dindel and SAMtools mpileup. [file 1471-2164-12-557-S5.BMP]

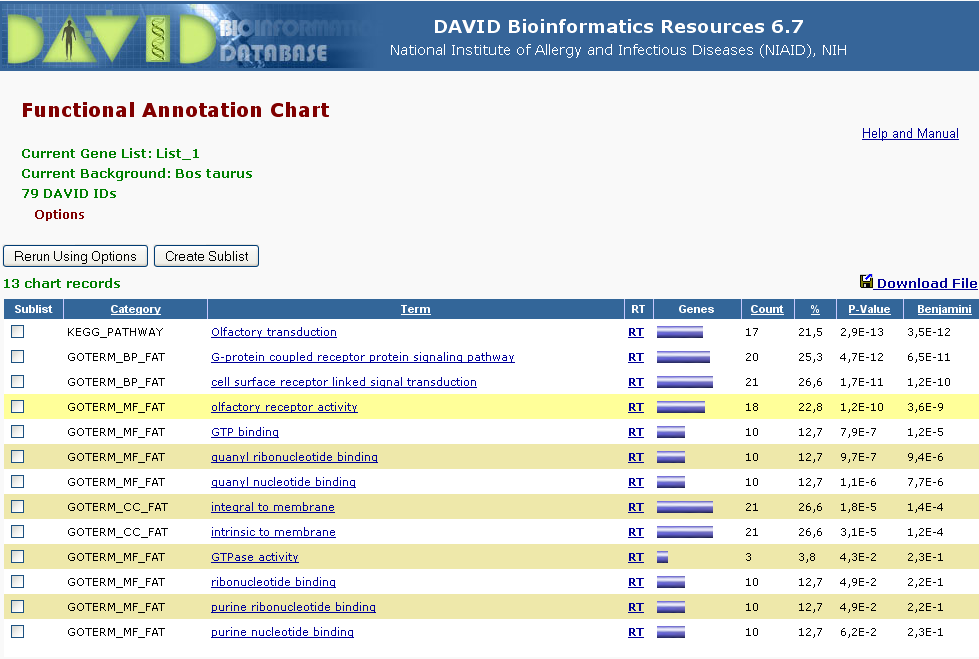

Supplement: Additional file 14 — Result of gene enrichment analysis. Gene and pathway enrichment for CNVs found using at least two platforms (DAVID database). [file 1471-2164-12-557-S14.BMP]
