# Supplementary material for: Global assessment of genomic variation in cattle by genome resequencing and high-throughput genotyping
Source: BMC Genomics. 2011 Nov 14;12:557. doi: 10.1186/1471-2164-12-557 (PMC3248099; doi:10.1186/1471-2164-12-557)
Supplement: Additional file 4 — Calculating description. A description of the methods used to calculate the BovineHD BeadChip detection rate and the mutation rate of the sequenced animal. [file 1471-2164-12-557-S4.PDF]

Calculation of BovineHD beadchip detection rate

With the assumption that SNP detection with both BovineHD beadchip and resequencing were random sampling, the (heterozygous) SNP detection rate of BovineHD beadchip was calculated as the percentage of heterozygous SNP (homozygous SNPs were excluded for calculating the detection rate because homozygous alleles identical to reference sequence were not listed by re-sequencing pipelines, and the rest of the homozygous SNPs have alternate allele to reference only count a small number) identified by resequencing that was also discovered by BovineHD beadchip. In order to improve the accuracy, 1392095 heterozygous SNPs identified by all the four resequencing pipelines (bwa + samtools, CLCBio Genomic Workbench, smalt + samtools, mosaik +gigaBayes) were used for this calculation. 131962 heterozygous SNPs were also detected by BovineHD beadchip, the detection rate of BovineHD beadchip was calculated as: 131962/1392095 = 0.095.

Calculation of mutation rate

The last heterozygous site called by both the BovineHD beadchip and the 4 resequencing pipelines with identical alleles in the largest IBD region may not be caused by any error but may be a real mutation. If we use this site as the real mutation detected by BovineHD beadchip, regarding calculated BovineHD SNP chip heterozygous detection rate in this study is 0.095. Then all possible mutations in this 90 Mb IBD region will be 1/0.095 =~11. And according to the pedigree of the sequenced **diploid** genome, the IBD region was inherited from a common ancestor three generations ago, this gives the haploid generation number as: diploid(2x) * 3 generation = 6. Therefore we may deduce the mutation rate as 1/0.095/2/3/90M = 1.9e-8.
